# Exploring medical student perceptions of a video-modified Peyton’s 4-step technique for teaching spinal and neurological examinations

**DOI:** 10.1080/10872981.2025.2519391

**Published:** 2025-06-16

**Authors:** Ramy Sherif, Ewan John, Michael John Haydon McCarthy

**Affiliations:** a Department of Spine, University Hospital of Wales, Cardiff University, Cardiff, UK; b School of Medicine, Cardiff University, Cardiff, UK; c Spinal department, University Hospital of Wales, Cardiff University, Cardiff, UK

**Keywords:** Peyton’s 4-step, video-based learning, clinical skills, neurological exams, medical education

## Abstract

**Background:**

Peyton’s 4-step technique is a highly recognized framework for teaching practical procedures. This study introduces a unique video-modified adaptation of the technique for teaching clinical examination and evaluates medical students’ perceptions, identifying key areas for enhancement.

**Methods:**

A cross-sectional study was conducted with 606 second-year medical students, utilizing an online questionnaire to assess their perceptions of the modified teaching method. Responses were measured using a 5-point Likert scale, with 354 students providing feedback.

**Results:**

Findings revealed significant improvements in student confidence following the video-modified examination session (p-value <0.05). The method was widely accepted by participants, who also provided constructive feedback for refining the teaching approach.

**Conclusions:**

The video-modified Peyton’s 4-step technique was perceived by students as a well-structured and engaging method for learning spinal and neurological examinations. Its adaptability to small group settings and integration of video-based instruction were well received and considered beneficial for understanding the procedural steps. While students reported increased confidence, this finding should be interpreted with caution given the cross-sectional design of the study. Incorporating this framework into medical curricula may enhance the delivery of clinical skills training, and future research should explore its impact through longitudinal and comparative studies.

## Introduction

Graduating medical students often lack confidence in performing neurological and spinal examinations, despite understanding their fundamental components. Many struggle with the skilled, nuanced aspects of these assessments and could benefit from more structured and frequent instruction. Teaching spinal, upper and lower neurological examinations is typically introduced early in the curriculum to prepare students for clinical placements. These skills are essential for accurate diagnosis and high quality patient care. However, ensuring proficiency in physical examination has become increasingly complex amid evolving curricula, reduced bedside teaching and advancements in healthcare technology. As such, there is a growing need to explore evidence based teaching strategies that improve both skill acquisition and long term retention [1–4].

One widely validated framework for teaching procedures skills is Peyton’s 4-step technique. This learner centered approach has been shown to enhance skill acquisition by providing structured guidance through sequential phases. The technique begins with the educator performing the entire procedure without interruption, followed by breaking the procedure into step by step components with detailed explanations. Students then verbalize the sequence of steps, ensuring comprehension, and finally, independently perform the procedure while receiving feedback for refinement and improvement. Peyton’s technique has been extensively validated in medical education, with a recent meta-analysis demonstrating its superiority over traditional methods, such as Halsted’s ‘see one, do one, teach one,’ which is often criticized for its lack of structure and variability in outcomes [5,6].

Video-based adaptations of Peyton’s technique have further enhanced its effectiveness, offering students dynamic, repeatable resources that align with modern learning preferences and cognitive load theory [7,8]. These video-based models provide learners with opportunities to repeatedly observe and practice skills, leading to improved motor skill retention, competence, and confidence [9,10].

Studies have demonstrated the superiority of this structured approach in various medical training contexts. Garg et al. (2023) found that students trained with Peyton’s method showed greater competency and confidence in performing fundamental clinical skills like blood pressure measurement and hand hygiene compared to traditional methods [11]. Similarly, Seifert et al. (2020) conducted a randomized controlled trial comparing video-based versions of Peyton’s and Halsted’s ‘see one, do one’ method for surgical skill acquisition, finding that Peyton’s approach significantly improved short term learning outcomes, particularly for complex procedural skills [12]. Additionally, Schleicher et al. (2021) explored a modified online adaptation of Peyton’s technique for teaching musculoskeletal and neurological examinations, reporting positive student engagement while acknowledging the limitations of remote learning for hands-on skills [13]. Collectively, these studies support Peyton’s 4-step technique, particularly in video based formats, as an effective and versatile model for clinical skill acquisition, surpassing traditional methods in many aspects.

Despite its growing application, limited research has explored student perceptions of Peyton’s technique, particularly in its video-based form. Understanding these perceptions is critical to refining the method and ensuring it aligns with student needs. In this study, we implemented a video-adapted version of Peyton’s 4-step technique to teach neurological and spinal examinations. Both qualitative and quantitative data were collected to evaluate student perceptions and identify opportunities for optimizing this instructional approach in medical education.

## Methods

### Study design

This cross-sectional study was conducted at a university medical school as part of a two-week educational module designed to teach spinal and neurological examination skills (Table 1). The standard guide used for tutor instruction was a structured institutional document developed by a committee of spinal surgeons to ensure consistency in teaching and assessment of neurological and spinal examination techniques. It was based on nationally recognized clinical guidelines, specifically the NICE Guideline NG59: *Low Back Pain and Sciatica in Over 16s – Assessment and Management* (2016, updated). The guide is regularly reviewed and updated to reflect current best practices, ensuring its continued relevance and minimizing the risk of outdated content impacting student learning [14,15].

**Table 1.** Case 14 low back pain course structure.

Week	Day	Session Description AM	Session Description PM
Week 1	Day 1	Lectures	Self-Directed Learning
Week 1	Day 2	Case-Based Learning	Case-Based Learning
Week 1	Day 3	Clinical skills*	Sport
Week 1	Day 4	Clinical skills* OR Community Placement/Pharmacy	Clinical skills* OR Community Placement/Pharmacy
Week 1	Day 5	Lectures	Anatomy**
Week 2	Day 6	Practical sessions and Tutorials/Anatomy**	Practical sessions and Tutorials/Anatomy**
Week 2	Day 7	Case-Based Learning/Anatomy**	Case-Based Learning
Week 2	Day 8	Clinical skills*	Sport
Week 2	Day 9	Clinical skills* OR Community Placement/Pharmacy	Clinical skills* OR Community Placement/Pharmacy
Week 2	Day 10	Case-Based Learning	Lectures

*Clinical skills consisted of half of the year in week 1 (150 students) split into 6 groups of 25 each receiving 90 mins clinical examination skills teaching delivered in groups of 6–8 by 4 tutors. The other half of the year (150 students) had community placements and Pharmacy sessions. This was reversed in week 2.

**Anatomy sessions were delivered in the anatomy centre cadaver lab 4 groups of 75 students each session lasting 180 mins.

The course was centered on a case-based learning approach using a clinical scenario involving a patient with lower back pain. This approach encouraged students to explore patient care through the biopsychosocial model, integrating biological aspects such as underlying pathology, psychological factors like mental wellbeing and patient beliefs, and social influences including family, work, and support systems. As second-year students had no prior formal training in neurological or spinal examinations, this cohort was well suited for evaluating the implementation of a structured teaching method. Their inexperience underscores the importance of selecting a framework known to support skill acquisition in novice learners. Supplementary lectures, tutorials, and practical sessions were integrated into the module to provide comprehensive support for case discussions and skill acquisition.

### Study participants

The study included 606 second-year undergraduate medical students enrolled in a five-year program during the 2022 and 2023 academic years. All students participated in the teaching sessions as part of the curriculum, but they had no prior formal training in conducting neurological or spinal examinations. Attendance at the sessions was mandatory; however, participation in the evaluation and feedback activities was entirely voluntary. Collection of feedback is standard practice at the University. As no demographic information was collected and all responses were anonymized, formal written consent was not required, and the study did not require specific ethical approval.

### Study intervention

The intervention employed a modified, video-based adaptation of Peyton’s 4-step technique as the instructional framework. Before the sessions, students were required to watch a series of videos demonstrating the key examination techniques. The videos provided both silent demonstrations of complete procedures and step-by-step deconstructions of the techniques, with explanations of the clinical reasoning behind each step. The preparatory material included a 27 minutes video on spinal history examination, 12 minutes video spinal examination techniques, an 8-minute video on the upper neurological examination and an 8-minute video on lower neurological examination [14].

In the practical sessions, students were divided into groups of six to eight, each facilitated by a single tutor. The clinical skills sessions were delivered across four designated days, which were strategically staggered over a two-week period. Students were allocated to attend on a rotational schedule, with half of the cohort participating in Week 1 and the remaining half in Week 2, to accommodate concurrent academic commitments such as community placements and pharmacy instruction. The initial phase involved group discussions guided by tutors, which reinforced the examination steps and the clinical rationale. These discussions aimed to enhance comprehension and consolidate the students’ understanding of the techniques for examining the upper and lower neurological systems and the spine. Subsequently, students paired up and performed the examinations on each other under the direct supervision of their tutor. Tutors provided immediate verbal feedback to refine students’ techniques and address any misunderstandings. A deliberate non-random pairing strategy was used to foster a comfortable and supportive learning environment. Tutors, drawn from the orthopedic and neurology departments, were provided with a standardized guide prior to the sessions to ensure consistency and alignment with the teaching objectives. The tutors were all asked to watch the videos.

### Evaluation of students’ perceptions

To evaluate the effectiveness of the intervention, an online questionnaire was administered to all participants after the sessions. The questionnaire utilized a 5-point Likert scale (1: Strongly disagree, 2: Disagree, 3: Neutral, 4: Agree, 5: Strongly agree) to assess key aspects of the sessions, including learning outcomes, clarity of instruction, perceived usefulness, enjoyment, and overall satisfaction. Additional items measured students’ confidence in performing neurological and spinal physical examinations, comfort with peer examination, and overall perceptions of the modified teaching structure.

The survey also included open-ended questions to capture qualitative insights into the most enjoyable aspects of the sessions and suggestions for improvement. This combination of quantitative and qualitative data provided a comprehensive assessment of students’ perceptions of the video-based Peyton’s 4-step technique and its application in teaching neurological and spinal examination skills.

### Quantitative analysis

Survey responses were analysed using the Statistical Package for the Social Sciences (IBM SPSS Statistics, Version 28; IBM Corp., Armonk, NY, USA). Descriptive statistics were applied, with Likert scale data summarized using means and standard deviations. A paired t-test was conducted to compare differences in confidence levels before and after the session, while an unpaired t-test assessed variations in session ratings between the 2022 and 2023 cohorts with statistical significance set at *p* < 0.05. Data processing and graphical representation were performed using Microsoft Excel. Free text responses were analysed using a semi quantitative thematic analysis combining structured content analysis with thematic coding. A structured approach was used to analyse responses, organizing them into predefined categories like usefulness, engagement, and areas for improvement, while also allowing new themes to emerge. Two researchers independently coded the data and resolved any differences through discussion. Expert instruction was provided by tutors, spinal consultants, and fellows, ensuring clinically relevant training and precise feedback to support student learning.

## Results

A total of 58% (354/606) of students responded to the online questionnaire. The findings indicate that the teaching sessions received positive ratings across all assessed domains in both 2022 and 2023. An unpaired t-test was conducted to assess differences between the two years, with a significance level of 0.05. The p-values for all domains were above this threshold, suggesting consistency in the positive feedback across both years.

**Figure 1.**
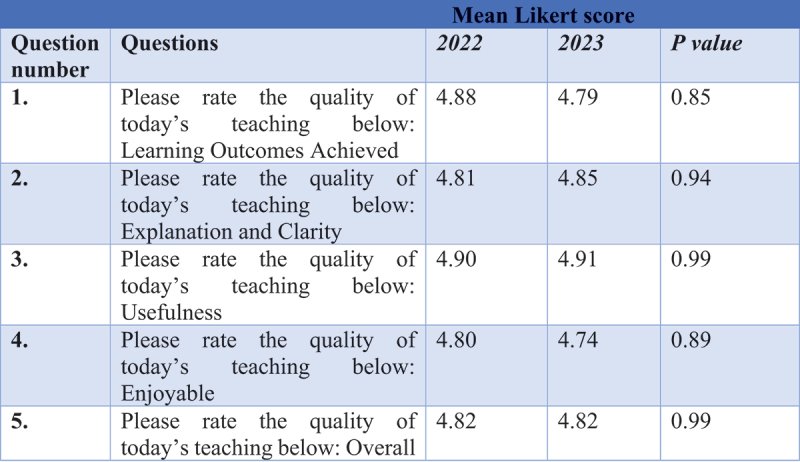
Responses of closed questions concerning students’ perceptions of the session domains across 2022 and 2023.

**Figure 2.**
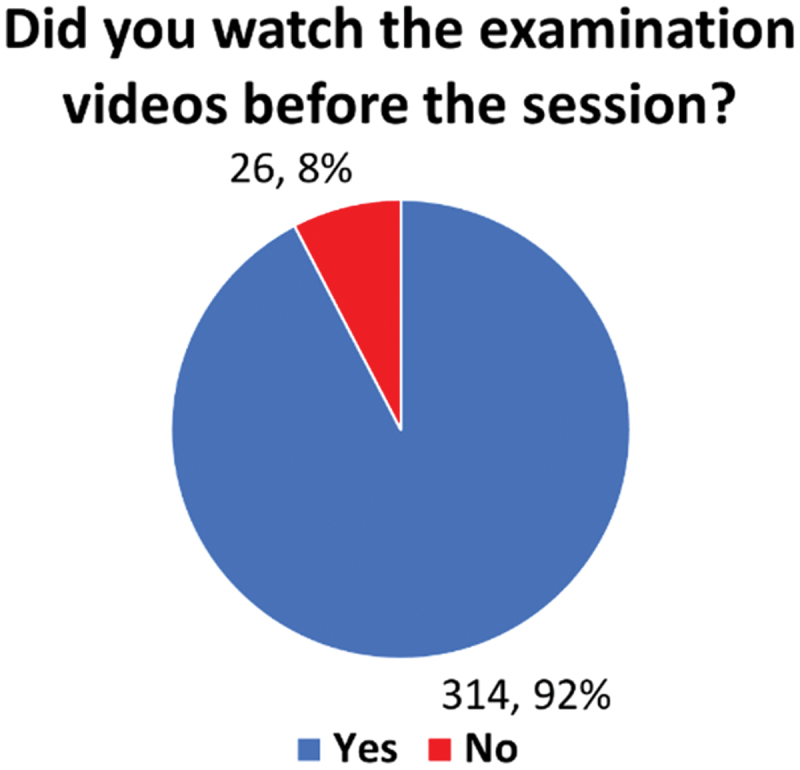
Yes/No responses of students’ in watching pre-session examination videos.

Further analysis of responses to closed-ended questions combined data from 2022 and 2023 after confirming, through an unpaired t-test, that there were no statistically significant differences between the years (*p* > 0.05) (Figure 1). This consistency validates the reliability of the findings. Figure 2 highlights students’ responses regarding the use of pre-examination videos, with 92% (314/340) indicating that they had accessed these videos prior to the sessions. Moreover, 91% (294/323) of students strongly agreed or agreed that the videos were a useful resource for preparation, showcasing the perceived utility and accessibility of the video-based materials.

**Figure 3.**
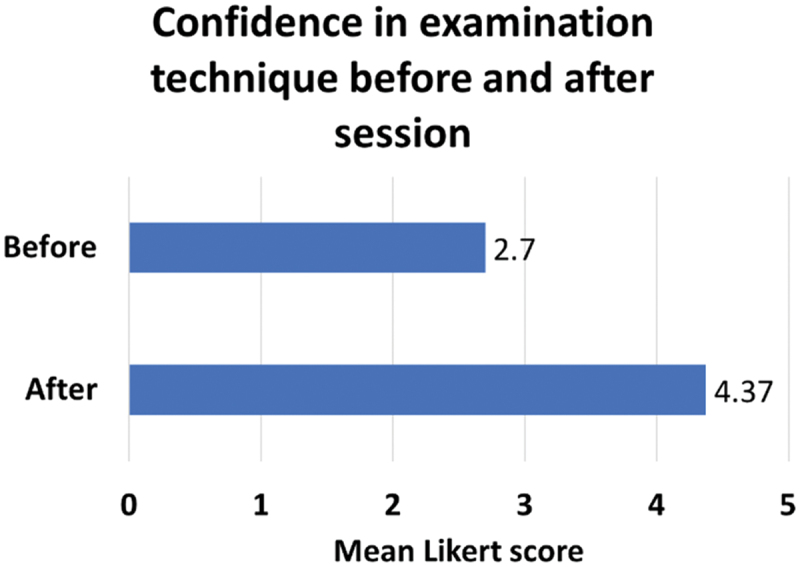
Students’ confidence in neurological and spinal examination before and after attending session.

The sessions also promoted a comfortable learning environment, as evidenced by 96% (312/326) of students reporting that they felt at ease performing peer examinations. Figure 3 demonstrates the significant improvement in students’ confidence in conducting neurological and spinal examinations. Before the session, the mean Likert score for confidence was 2.70, reflecting limited confidence in their techniques. After the session, this score rose to 4.37. A paired t-test revealed a statistically significant difference between pre- and post-session confidence levels (*p* < 0.0001), suggesting that the combination of video-based instruction and supervised hands-on practice was effective in enhancing confidence.

**Figure 4.**
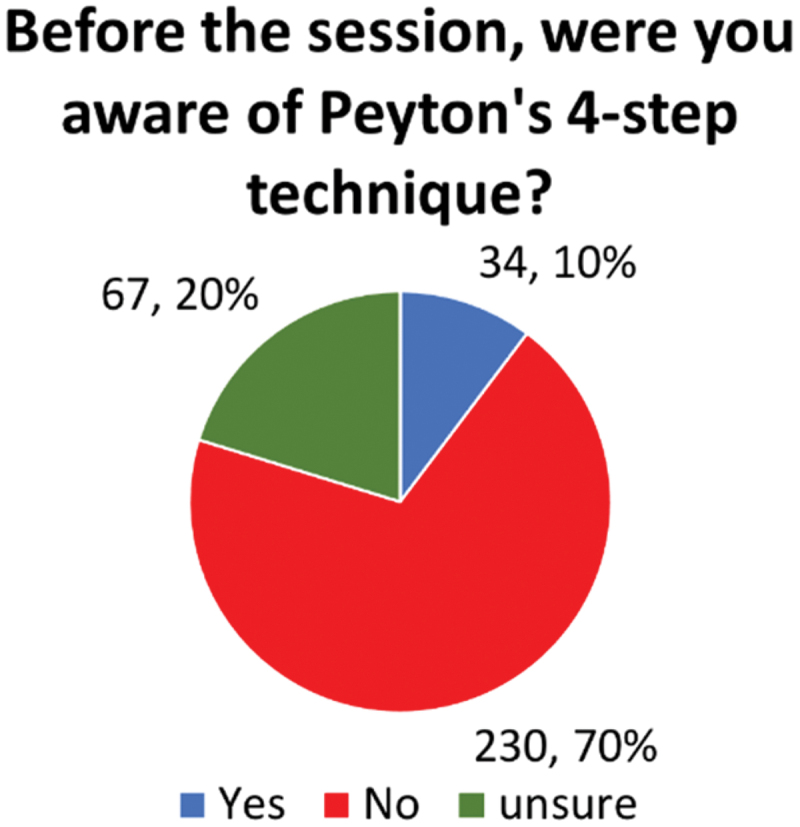
Yes/No/Unsure responses of students to their awareness of Peyton’s 4-step technique.

**Figure 5.**
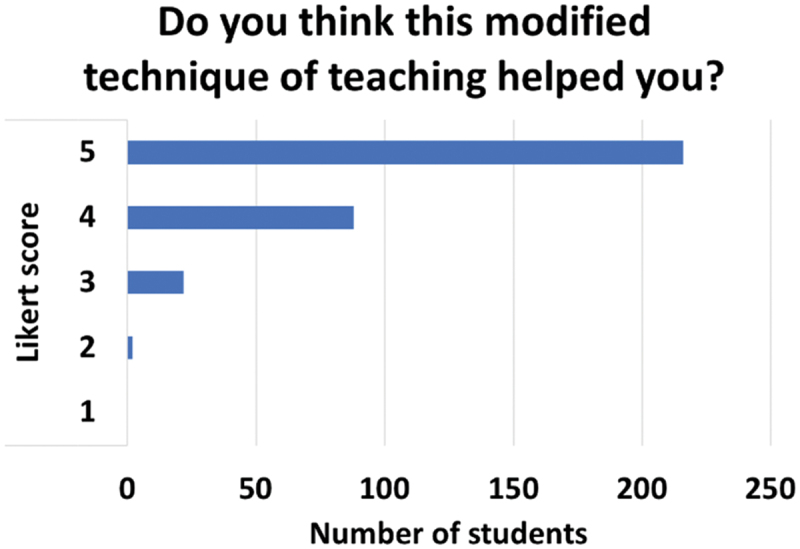
Students’ perceptions of the video-modified Peyton’s 4-step technique.

While the majority of students were unfamiliar with Peyton’s 4-step technique before the session (90%, 297/331) (Figure 4), they highly valued the modified approach used in the teaching sessions. Figure 5 shows a mean Likert score of 4.58 for the perceived helpfulness of the modified teaching method, indicating strong acceptance among students. Qualitative feedback further emphasized the positive reception, with recurring themes highlighting the practical, interactive, and supportive nature of the sessions.

Students appreciated the opportunity to engage in practical, hands-on learning with peers, which they felt was essential for improving their examination techniques. Many commented on how the relaxed environment allowed them to practice comfortably without fear of judgment. Small group settings were also praised for reducing individual pressure and fostering active participation. Students noted that these smaller groups facilitated more opportunities for practice and personalized feedback from tutors, which helped them identify and correct errors effectively.

The teaching strategies employed during the sessions were highly regarded. The use of pre-session videos was particularly valued, with students stating that these materials provided a clear structure for the examinations and increased their confidence. Many students described the teaching sessions as among the most effective they had experienced during their medical education, particularly appreciating the demonstrations, mnemonics, and systematic approach used to deliver the material.

The tutors also received significant praise for their approachability, encouragement, and ability to provide constructive feedback. Students highlighted how immediate feedback from tutors helped them refine their skills and gain confidence during the session. The supportive attitude of the tutors contributed to a positive learning environment that motivated students to engage actively in the exercises.

Despite the overwhelmingly positive feedback, students suggested several improvements to enhance the sessions further. Some noted that the sessions felt slightly rushed, with insufficient time for everyone to practice all components of the examination thoroughly. They suggested extending the session by 30 minutes to ensure that all students had the opportunity to perform each examination. Additionally, students expressed interest in receiving printed materials, such as detailed examination instructions and ASIA charts, to supplement their learning and provide additional resources for revision. A few students recommended incorporating real patients into the sessions to simulate a more authentic clinical environment, which they felt would provide invaluable experience.

Overall, the results demonstrate the effectiveness of the modified teaching sessions in improving students’ confidence and skills. While students were highly satisfied with the sessions, the suggested adjustments such as extending the duration and providing supplementary materials could further enhance the learning experience and better prepare students for clinical practice.

## Discussion

The purpose of this study was to explore student perceptions of the video-modified Peyton’s 4-step technique in teaching spinal and neurological examinations. Previous research has consistently demonstrated the superiority of Peyton’s approach over traditional methods like The Halsted’s method in enhancing procedural skills, particularly for complex tasks [5]. This study reinforces these findings, showing that the video-modified technique was well-received by students and significantly boosted their confidence in performing examinations. Notably, 92% of participants confirmed they had used the preparatory videos, underscoring both the accessibility and value of this resource in supporting learning.

Although Peyton’s technique was originally developed for a 1:1 student-to-teacher ratio [6], this study implemented the method in small groups of 6–8 students per tutor. This approach is consistent with existing evidence suggesting that Peyton’s technique can be effectively applied in small-group settings with fewer than nine students per tutor [2]. However, some students expressed concerns about not having sufficient opportunities to practice all examinations during the session. Addressing this issue could involve extending session durations or further reducing the student-to-teacher ratio to allow for more individualized practice.

Although Peyton’s method is recognized for its instructional value, it remained unfamiliar to many students prior to the session, highlighting a gap in awareness of evidence-based teaching strategies and suggesting its potential as a valuable addition to current methods for teaching neurological and spinal examinations. Given the critical role of education in healthcare professionals’ careers, incorporating structured teaching frameworks like Peyton’s into broader medical curricula could prepare future educators to implement effective, evidence-based instructional methods [8]. This would ensure that high-quality teaching practices are consistently propagated across generations of healthcare providers.

Peer observation, a key component of this study, was integrated into the performance phase of Peyton’s technique, with 96% of students expressing comfort in examining their peers. Research supports the benefits of peer observation in enhancing both skill acquisition and retention [6]. Allowing students to choose their own peers contributed to a more relaxed learning environment, further improving engagement and comfort. Additionally, the small-group format was particularly appreciated, as it reduced pressure while providing more opportunities for hands-on practice and individualized feedback.

The effectiveness of Peyton’s Four-Step Approach has been further substantiated by multiple studies in clinical skills training across various medical disciplines. Krautter et al. (2011) found that this method not only improved professionalism and doctor-patient communication but also reduced the time required for procedural performance [16]. Giacomino et al. (2020) conducted a systematic review and meta-analysis, confirming that Peyton’s approach enhances skill acquisition, particularly in settings with small student-teacher ratios [17]. A study by Nikendei et al. (2014) modified the method for small-group teaching, demonstrating its feasibility and effectiveness in fostering skill retention [18]. Furthermore, Gradl-Dietsch et al. (2016) applied Peyton’s method to spinal manipulation training and reported superior skill acquisition compared to conventional instruction, though skill retention declined over time [19]. Collectively, these findings reinforce the value of Peyton’s approach as an effective instructional model, particularly when implemented in structured, small-group settings that optimize learning and engagement.

## Areas for improvement

While the modified Peyton’s technique was highly effective, this study identified several areas for refinement to optimize learning outcomes. Encouraging students to engage in mental repetition of examination steps during the session could improve retention and confidence. Studies suggest that mental rehearsal enhances procedural understanding and memory consolidation [8]. Introducing anatomy instruction relevant to the examinations prior to the sessions may also provide a stronger foundation for understanding the physical examination techniques, as research indicates that anatomy integration improves comprehension [10].

Incorporating clinical cases into the teaching sessions may further contextualize the examination techniques, improving students’ preparedness for clinical placements. Although evidence on this approach in physical examination training is limited, studies on preclinical curricula suggest that case-based learning is highly valued by students and reduces their stress when transitioning to clinical environments [20–22].

Student feedback also included practical suggestions such as extending the session duration, providing additional learning materials like printed guides and mnemonics, and incorporating real patients into the training. While these enhancements could improve authenticity, confidence, and skill retention, we face challenges such as time constraints, limited lecture space, and tutor availability especially with the growing number of medical students, which is expected to reach 340 in 2024. To address these limitations and optimize learning, we developed this program as a structured and efficient approach to improving clinical skills training.

## Limitations and future direction

This study has limitations, including selection bias from a 58% response rate, potential social desirability bias from self reported confidence levels and limited generalizability as it focused on second year medical students at a single institution. The large sample size (354 respondents) strengthens the reliability of the findings and mitigates selection bias as it represents a substantial proportion of the cohort. The absence of a comparison cohort using classical teaching methods prevents assessing the relative effectiveness of the video-modified Peyton’s technique. Future research should incorporate comparative studies and objective skill assessments to better evaluate its impact and ensure alignment between perceived and actual performance.

## Conclusion

The video-modified Peyton’s 4-step technique was perceived by students as a well-structured and engaging method for learning spinal and neurological examinations. Its adaptability to small group settings and integration of video-based instruction were well received and considered beneficial for understanding the procedural steps. While students reported increased confidence, this finding should be interpreted with caution given the cross-sectional design of the study. Incorporating this framework into medical curricula may enhance the delivery of clinical skills training, and future research should explore its impact through longitudinal and comparative studies.
